# Opposite Roles of Wnt7a and Sfrp1 in Modulating Proper Development of Neural Progenitors in the Mouse Cerebral Cortex

**DOI:** 10.3389/fnmol.2018.00247

**Published:** 2018-07-17

**Authors:** Nan Miao, Shan Bian, Trevor Lee, Taufif Mubarak, Shiying Huang, Zhihong Wen, Ghulam Hussain, Tao Sun

**Affiliations:** ^1^Center for Precision Medicine, School of Medicine and School of Biomedical Sciences, Huaqiao University, Xiamen, China; ^2^Department of Cell and Developmental Biology, Weill Cornell Medicine, Cornell University, New York, NY, United States; ^3^College of Oceanology and Food Science, Quanzhou Normal University, Quanzhou, China; ^4^Marine Biomedical Laboratory and Center for Translational Biopharmaceuticals, Department of Marine Biotechnology and Resources, National Sun Yat-sen University, Kaohsiung, Taiwan; ^5^Department of Physiology, Government College University, Faisalabad, Pakistan

**Keywords:** Wnt7a, Sfrp1, cerebral cortex, neural progenitors, antagonist

## Abstract

The Wingless (Wnt)-mediated signals are involved in many important aspects of development of the mammalian cerebral cortex. How Wnts interact with their modulators in cortical development is still unclear. Here, we show that Wnt7a and secreted frizzled-related protein 1 (Sfrp1), a soluble modulator of Wnts, are co-expressed in mouse embryonic cortical neural progenitors (NPs). Knockout of Wnt7a in mice causes microcephaly due to reduced NP population and neurogenesis, and Sfrp1 has an opposing effect compared to Wnt7a. Similar to Dkk1, Sfrp1 decreases the Wnt1 and Wnt7a activity *in vitro*. Our results suggest that Wnt7a and Sfrp1 play opposite roles to ensure proper NP progeny in the developing cortex.

## Introduction

During development of the mammalian CNS, billions of neurons are produced from proliferating NPs (Rakic, 2009). In the cerebral cortex, NPs are expanded through symmetric and asymmetric division at the VZ and SVZ (Haubensak et al., 2004; Gotz and Huttner, 2005; Homem et al., 2015). The proper control of proliferation, survival and differentiation of NPs is the key step for normal cortical formation (Rakic, 2007, 2009; Zhao et al., 2008; Sun and Hevner, 2014).

A number of signaling pathways that regulate the switch and balance between proliferation and differentiation of NPs have been defined, including the Notch, Sonic hedgehog, fibroblast growth factor, TGF-β/Smads, and Wnt pathways (Chenn and Walsh, 1999; Rowitch et al., 1999; Hirabayashi et al., 2004; Joksimovic et al., 2009; Aguirre et al., 2010; Menendez et al., 2011; Rash et al., 2011). Wnt signaling pathways play crucial roles in neurogenesis (Kuwabara et al., 2009; Durak et al., 2016). For example, the canonical Wnt/β-catenin pathway is required for NP self-renewal and differentiation (Chenn and Walsh, 2003; Kalani et al., 2008; Bengoa-Vergniory et al., 2014; Delaunay et al., 2014; Bengoa-Vergniory and Kypta, 2015; Garriock et al., 2015). Among the Wnt signaling molecules, *Wnt7a* has been shown to be critical in axonal remodeling, guidance, synaptogenesis and neurotransmitter release in the hippocampus (Hall et al., 2000; Cerpa et al., 2008; Ciani et al., 2011, 2015). *Wnt7a* controls neurogenesis through regulating genes involved in both cell cycle control and neuronal differentiation (Qu et al., 2013; Long et al., 2016).

Furthermore, three distinct receptor families have been reported to mediate the Wnt signaling: Fz, RoR, and Ryk (van Amerongen et al., 2008; Angers and Moon, 2009). In the nervous system, Fzs regulate a range of functions from neuronal differentiation to cell polarity, axon guidance, and cell survival (Van Raay et al., 2005; Prasad and Clark, 2006; Liu et al., 2008; Kilander et al., 2014; Zhou and Nathans, 2014; Morello et al., 2015; Chailangkarn et al., 2016). Moreover, Sfrps are a family of secreted factors that modulate Wnt-induced β-catenin pathway through selectively sequestering specific Wnts in different neurons by possessing the Wnt-binding frizzled CRD (Dann et al., 2001; Bovolenta et al., 2008; Nathan and Tzahor, 2009; Lavergne et al., 2011). For example, both *Sfrp1* and *Sfrp2* can be the dominant negative inhibitors of *Wnt3a* to inhibit proliferation in the developing chick neural tube (Galli et al., 2006), and *Sfrp2* can negatively regulate the Wnt signaling in the CNS of *Pax6* mutant mice via inhibiting *Wnt7b* (Kim et al., 2001a). *Sfrp1* knockout mice display abnormal cortical morphogenesis (Esteve et al., 2018). However, the precise regulation of Wnts and their antagonist Sfrps in mammalian cortical neurogenesis is still unclear.

In this study, we show that *Wnt7a* and *Sfrp1* are co-expressed in the VZ of mouse embryonic cerebral cortices. Knockout of *Wnt7a* causes microcephaly due to reduced numbers of NPs and decreased neurogenesis. *Sfrp1* showed overexpression leads to a decrease in the NP population. Similar to the known Wnt antagonist *Dkk1*, *Sfrp1* directly blocks the *Wnt1* and *Wnt7a* activity *in vitro*. Our results indicate that opposite effects of Wnt7a and Sfrp1 play an important role in expansion of cortical NPs.

## Materials and Methods

### Animals and Genotyping

The *Wnt7a* knockout mice (*Wnt7a* KO, *Wnt7a^-/-^*) were generated by mating female *Wnt7a* heterozygous mice (*Wnt7a^+/-^*) with male *Wnt7a* heterozygous mice (*Wnt7a^+/-^*). Mice that only have the mutant allele (*Wnt7a^-/-^*) were *Wnt7a* KO mice, wild-type (WT) mice were used as controls. To achieve knockout of *Wnt7a*, a double-selection gene-replacement construct was designed to insert a neo gene into a Nael site in the second exon of the *Wnt7a* gene (Parr and McMahon, 1995; Ashrafi et al., 2012).

For staging of embryos, midday of the day of vaginal-plug formation was considered as E0.5; the first 24 h after birth were defined as P0. Animal use was overseen by the Animal Facility at Weill Cornell Medical College (Protocol number #2011-0062), and was performed according to the institutional ethical guidelines for animal experiments.

Mouse tail-tip biopsies were used for genotyping by PCR reactions using the following primers: for *Wnt7a* KO, forward: 5-CTCTTCGGTGGTAGCTCTGG-3 and reverse-1: 5-TCACGTCCTGCACGACGCGAGCTG-3 (product size: 350 bp); for WT, reverse-2: 5-TCCTTCCCGAAGACAGTACG-3 (product sizes: 560 bp).

### RNA Sequencing (RNA-Seq)

Total RNAs for RNA-seq were extracted from three individual E12.5 mouse cerebral cortices using TRIzol (Invitrogen, United States) according to manufacturer’s instructions. The ribosome RNA (rRNA) removal, generation of cDNA library and high-throughput sequencing were performed on the Ion proton platform (Life Technologies, United States) from the NovelBio Bio-Pharm Technology Company (Shanghai, China). Three sets of raw reads were obtained, and data were deposited in Gene Expression Omnibus (GEO^1^) under the series number GSE116056. After removing contaminated and low-quality sequences, all reads were mapped onto the Ensembl mouse reference genome. Gene expression level were calculated by RPKM (reads per kilo-bases per million mapped reads).

### *In Situ* Hybridization

*In situ* hybridization was performed as described: following fixation with 4% PFA, acetylation with acetylation buffer (1.3% triethanolamine, 0.25% acetic anhydride, 20 mM HCl), treatment with proteinase K (5 μg/ml, IBI Scientific) and pre-hybridization (1 × SSC, 50% formamide, 0.1 mg/ml Salmon Sperm DNA Solution, 1 × Denhart, 5 mM EDTA, pH 7.5), brain sections were hybridized with DIG-labeled LNA probes at Tm -22°C overnight. After washing with pre-cooled wash buffer (1 × SSC, 50% formamide, 0.1% Tween-20) and 1 × MABT, sections were blocked with blocking buffer (1 × MABT, 2% blocking solution, 20% heat-inactived sheep serum) and incubated with anti-DIG antibody (1:1, 500, Roche) at 4°C overnight. Brain sections were washed with 1 × MABT and Staining buffer (0.1 M NaCl, 50 mM MgCl_2_, 0.1 M Tris-HCl, pH 9.5), stained with BM purple (Roche) at room temperature until ideal intensity was reached. The antisense RNA probe (*Sfrp1*, *Wnt7a*, *Wnt7b*, *Pax6, Ngn2*, and *Hes5*) was labeled using the DIG RNA labeling Kit (Roche, Switzerland) following the manufacturer’s instructions.

### Nissl Staining and Measuring Brain Size

Brain sections (14 μm) were processed through incubation in the subsequent solutions in the following order: ethanol/chloroform (1:1, overnight), 100% ethanol (30 s), 95% ethanol (30 s), distilled water (30 s, twice), cresyl violet (3–5 min), distilled water (2 min, three times), 50% ethanol (2 min), 95% ethanol (5–30 min), 100% ethanol (5 min, twice), xylene (3 min, twice). Thereafter, the sections were mounted with a coverslip.

The *Wnt7a* KO and WT brain images were captured in one picture, and the thickness of the cortex and CP was measured separately. The relative thickness of the cortex and CP in the KO was normalized from dividing the mean length of KO by that of the WT groups. At least three brains, and two chosen areas in each brain section were measured and averaged in each group. All data are presented as mean ± SEM. *P*-values were calculated using unpaired Student’s *t*-test.

### RNA and qRT-PCR

The RNAs for RT-PCR from five stages of samples (E12.5, E13.5, E14.5, E15.5, and E17.5), were extracted by TRIzol (Invitrogen, United States), with three mouse cerebral cortices from each age group. Experimental protocols of embryo treatment used here were approved by Weill Cornell Medical College’s animal care and use committee. The procedures were carried out in accordance with the approved guidelines. After RNA extraction, the cDNA for RT-PCR was synthesized using high-capacity cDNA Reverse Transcription kit (Applied Biosystems). The qRT-PCR reactions were carried out in the Bio-Rad CFX-384 system, using the reaction mixture SYBR Green Mix (Bio-Rad, United States) with the aforementioned cDNA samples.

β-Actin was used as an internal control, and was used to normalize the relative mRNA expression level. Each group had three biological repetitions, and all experiments were performed in triplicate, and each experiment was repeated at least twice. The qRT-PCR primers are: *Wnt7a*, forward: 5′-CCGAAATGGCCGTTGG-3′ and reverse: 5′-CGATGCCGTAGCGGATGT-3′ (PCR product: 251 bp); *Sfrp1*, forward: 5′-CAACGTGGGCTACAAGAAGAT-3′ and reverse: 5′-GGCCAGTAGAAGCCGAAG AAC-3′ (product size: 249 bp); β-actin, forward: 5′-GGCTGTATTCCCCTCCATCG-3′ and reverse: 5′-CCAGTTGGTAACAATGCCATGT-3′ (product size: 245 bp). All data are presented as mean ± SEM. *P-*values were calculated using unpaired Student’s *t*-test.

### Tissue Preparation, Immunohistochemistry, and Analysis

Immunohistochemistry was performed as described: mouse brains were fixed in 4% PFA in phosphate-buffered saline (PBS) over night, incubated in 25–30% sucrose in PBS, embedded in OCT and stored at -80°C until use. Brains were sectioned (14–16 μm) using a cryostat. For antigen recovery, sections were incubated in heated (95–100°C) antigen recovery solution (1 mM EDTA, 5 mM Tris, pH 8.0) for 15–20 min, and cooled down for 20–30 min. Before applying antibodies, sections were blocked in 10% normal goat serum (NGS) in PBS with 0.1% Tween-20 (PBT) for 1 h. Sections were incubated with primary antibodies at 4°C overnight and visualized using goat anti-rabbit IgG–Alexa-Fluor-488 and/or goat anti-mouse IgG–Alexa-Fluor-546 (1:300, Molecular Probes) for 1.5 h at room temperature. Images were captured using a Leica digital camera under a fluorescent microscope (Leica DMI6000B) or a Zeiss confocal microscope.

The following antibodies were used: bromodeoxyuridine (BrdU) (1:50, DSHB), Ki67 (1:500, Abcam), Pax6 (1:30, DSHB), Tbr1 (1:2500, Abcam), Tbr2 (1:2000, kindly provided by Robert Hevner, University of Washington, Seattle, WA, United States), Ctip2 (1:1000, Abcam), Satb2 (1:1000, Abcam), GFP (1:600, DAKO), Neun (1:300, Chemicon), Wnt7a (1:1000, Abcam) and Sfrp1(1:1000, Abcam).

Cell counting in the mouse brain sections was performed on a fixed width (200 μm bin) of a representative column in the cortical wall. All sections analyzed were selected from a similar medial point on the anterior-posterior axis. Cell counting was performed in minimal three chosen areas in each brain, and at least three brains were analyzed in each group. Cell counting in each chosen area was repeated at least three times and a mean was obtained. All data are presented as mean ± SEM. *P*-values were calculated using unpaired Student’s *t*-test.

### Plasmid DNA Constructs

To clone *Sfrp1*, *Dkk1* and *Wnt7a* coding sequences into *pCAGIG* for IUE, *Sfrp1*, *Dkk1* and *Wnt7a* coding sequences from *pGEM-T* was attached to d2EGFP, a destabilized variant of the wild-type GFP, and then subcloned *d2EGFP-Sfrp1*, -*Dkk1* and -*Wnt7a* coding sequence fragments into *pCAGIG*.

Full length coding sequences (CDSs) for *Sfrp1*, *Dkk1* and *Wnt7a* were cloned using the following primers: *Sfrp1*, forward: 5′-ATTCCGCTCGAGCGGGTCGCCGAGCAACATGGGCGTC-3′ and reverse: 5′-ATTCCTTAAGGCCTTCCCCAGTCCGCCCCAG-3′ (PCR product: 954 bp); *Wnt7a*, forward: 5′-GCACTCGAGCAGCGGGGACTATGACCCGGAAAGCGC-3′ and reverse: 5′-CATTCACTTGCACGTATACATCTCCGTG-3′ (PCR product: 1,053 bp); *DKK1*, forward: 5′-CGGAATTCGGAGATGATGGTTGTGTGTGC-3′ and reverse: 5′-GGTTTAGTGTCTCTG GCAGGTGTG-3′ (PCR product: 826 bp).

The *Sfrp1*, *Dkk1* coding sequences were subcloned into the *pcDNA3.1* vector for the *TOPflash* and *FOPflash luciferase* reporter (Promega, United States) assay.

### RNA Interference Design and Efficiency Analysis

To knockdown *Sfrp1*, 4 different *Sfrp1* specific *short hairpin RNA* (*Sfrp1-shRNA*) were designed and cloned into the *pSilencer* vector, separately. To analyze interference efficiency, Neuro2A cells were plated into 6-well plates in triplicate, and were transfected with four *Sfrp1-shRNA* using Lipofectamine 3000 (Invitrogen, United States). Cells were cultured for 2 days and the endogenous *Sfrp1* knockdown efficiency was verified by qRT-PCR. The *shRNA* with the highest knockdown efficiency was selected to perform further IUE in cerebral cortices.

The following oligos were used to clone *Sfrp1-shRNA*: *Sfrp1-shRNA1*, 5′-CACCGCTACAAGAAGATGGTGCTGCTTCAAGAGAGCAGCACCATCTTCTGGTAGCTTTTTTG-3′ (Target site: GCTACAAGAAGATGGTGCTGC, 498–519); *Sfrp1-shRNA2*, 5′-CACCGCCACAACTTTCTCATCATGGTTCAAGAGACCATGATGAGAAAGTTGTGGCTTTTTTG-3′ (Target site: GCCACAACTTTCTCATCATGG, 1,077–1,098); *Sfrp1-shRNA3*, 5′-CACCGCCATTCACAAGTGGGACAAGTTCAAGAGACTTGTCCCACTTGTCCCACTTGTGAATGGCTTTTTTG-3′ (Target site: GCCACAACTTTCTCATCATGG, 1,130–1,151); *Sfrp1-shRNA4*, 5′-CACCGCAGTTCTTCGGCTTCTACTGTTCAAGAGACAGTAGAAGCCGAAGAACTGCTTTTTTG-3′ (Target site: GCAGTTCTTCGGCTTCTACTG, 715–736); for negative control, 5′-CACCGTTCTCCGAACGTGTCACGTTTCAAGAGAACGTGACACGTTCGGAGAATTTTTTG-3′.

### *In Utero* Electroporation

*In utero* electroporation was performed in E12.5 embryos according to the published protocol (Saito and Nakatsuji, 2001; Saito, 2006; Ito et al., 2014). Briefly, plasmid DNA was prepared using the EndoFree Plasmid Maxi Kit (Qiagen) according to manufacturer’s instructions, and diluted to 2 μg/μl. DNA solution was injected into the lateral ventricle of the cerebral cortex, and electroporated with five 50-ms pulses at 35V using an ECM830 electro square porator (BTX). Embryos were allowed to develop to E13.5. Animals with their brains electroporated, as detected by the GFP fluorescence under a fluorescent dissection scope (Leica, MZ16F), were selected for further analyses. Cell counting was performed in minimal three chosen areas in each brain, and at least three electroporated brains for each construct were analyzed. Cell counting in each chosen area was repeated at least three times and a mean was obtained.

### *TOPflash* and *FOPflash Luciferase* Reporter Assay

The coding sequences of the *Wnt7a* and *Wnt1* were amplified by PCR from mouse cDNA. Reporter genes were cloned into *TOPflash* and *FOPflash* vector (Promega, United States). For transfections, mouse Neuro2A cells were suspended in DMEM and plated into 24-well plates in triplicate at 1.5 × 10 ^4^cells/100 mL. Adherent cells were co-transfected with 100 ng/mL *luciferase* reporter containing the reporter gene and 60 ng/mL vector (pcDNA3.1 blank vector, pcDNA3.1-*Dkk1* and pcDNA3.1-*Sfrp1*) using Lipofectamine 3000 (Invitrogen, United States). After 48 h, cells were harvested and *luciferase* activity was measured using the *luciferase* reporter assay system (Cat. #E1910, Promega, United States) according to the manufacturer’s protocol.

The relative *luciferase* activity was normalized from the mean of pcDNA3.1 blank vector, separately. Each group had three biological repetitions, and experiments were performed in triplicate and each sample was repeated at least three times. All results are presented as mean ± SEM. *P-*values were calculated using unpaired Student’s *t*-test.

### Statistical Analysis

All experiments using cultured cells and mouse embryos were repeated at least with three biological replicates. All results are presented as mean ± standard error of the mean (SEM). *P*-values were determined by unpaired Student’s *t*-test for assessing the significance of differences between two treatments (See each figure for details). *P-*values <0.05 were considered significant. Significant differences were denoted as ^∗^*P-*values < 0.05, ^∗∗^*P-*values < 0.01, ^∗∗∗^*P-*values < 0.001.

## Results

### *Wnt7a* and *Sfrp1* Are Co-expressed in NPs in the VZ

To screen genes that are highly expressed in the mouse E12.5 cerebral cortices, RNA sequencing (RNA-seq) was performed. 30,827,078 and 29,345,746 and 32,038,052 raw sequencing reads, and 28,547,544 and 27,289,172 and 29,753,653 clean reads, respectively, were obtained from three individual E12.5 cortices (Supplementary Table S1). The mapping rates of clean reads are 92.2%, 93.4%, and 92.6% (Supplementary Table S2). Among these genes, *Wnt7a*, *Wnt7b*, and *Sfrp1* showed high expression (RPKM >500) (Supplementary Figure S1A and Supplementary Table S4). Moreover, *Wnt7b*, *Wnt7a*, and *Wnt5a* displayed higher abundant expression levels than other Wnt genes (Supplementary Tables S3, S4).

**FIGURE 1:**
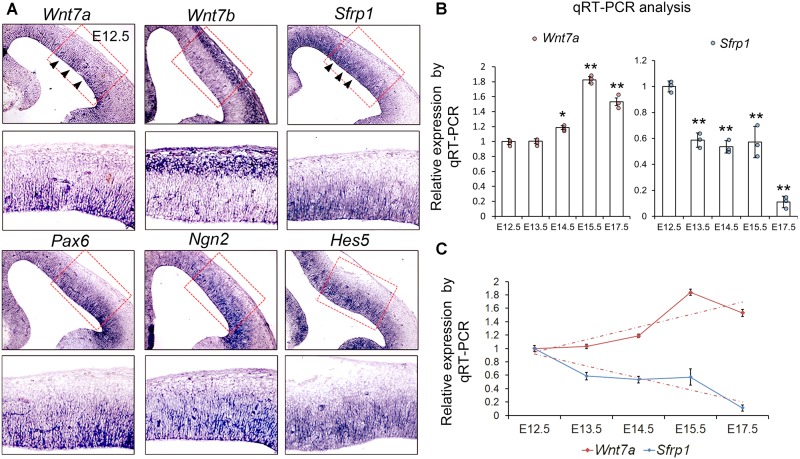
*Wnt7a* and *Sfrp1* are co-expressed in neural progenitors and show opposite expression trends. **(A)** In coronal sections of mouse E12.5 cerebral cortices, *Wnt7a*, *Sfrp1*, *Pax6*, *Ngn2*, and *Hes5* were expressed in the ventricular zone (arrowheads). Conversely, *Wnt7b* was expressed in newborn neurons. Red boxes show high power views. **(B)** qRT-PCR analysis of *Wnt7a* and *Sfrp1* expression levels at different embryonic stages (E12.5, E13.5, E14.5, E15.5, and E17.5). All comparisons were made with that of values at E12.5. Values of histogram represent mean ± SEM, and each dot represents a data point in each biology repeat (*n* = 3, ^∗^*P* < 0.05; ^∗∗^*P* < 0.01; unpaired Student’s *t*-test). **(C)** Opposite expression trends between *Wnt7a* and *Sfrp1* at different embryonic stages (from E12.5 to E17.5) measured by qRT-PCR.

To verify the RNA-seq data, we examined expression patterns of *Wnt7a*, *Wnt7b*, and *Sfrp1*, and compared them with those of NP markers such as *Pax6*, *Ngn2*, and *Hes5*, and other *Sfrp*s such as *Sfrp2, Sfrp4*, and *Sfrp5* in the mouse cortex at E12.5 using ISH (**Figure 1A** and Supplementary Figure S1B). We found that both *Wnt7a* and *Sfrp1* are expressed in the VZ of the E12.5 cortex (**Figure 1A**). Moreover, expression of *Wnt7a* and *Sfrp1* was co-localized with that of *Pax6*, *Ngn2* and *Hes5*, suggesting that *Wnt7a* and *Sfrp1* are largely expressed in NPs (**Figure 1A**). Conversely, *Wnt7b* was highly expressed in newborn neurons, and other *Sfrp*s such as *Sfrp2* displayed low expression in the cortex (**Figure 1A** and Supplementary Figure S1B).

Next, we investigated whether expression levels of *Wnt7a* and *Sfrp1* progressively change over embryonic stages at E12.5, E13.5, E14.5, E15.5, and E17.5 using qRT-PCR. *Wnt7a* displayed ascending expression from E12.5 to E15.5 (**Figure 1B**). *Sfrp1* expression showed a gradual decline from E12.5 to E17.5 (**Figure 1C**). Compared to *Wnt7a*, *Sfrp1* displays overlapping expression with *Wnt7a* in the VZ and opposite expression levels, implying distinct roles of Wnt7a and Sfrp1 in cortical development.

### *Wnt7a* Positively Regulates Proliferation of NPs and Promotes Neurogenesis

**FIGURE 2:**
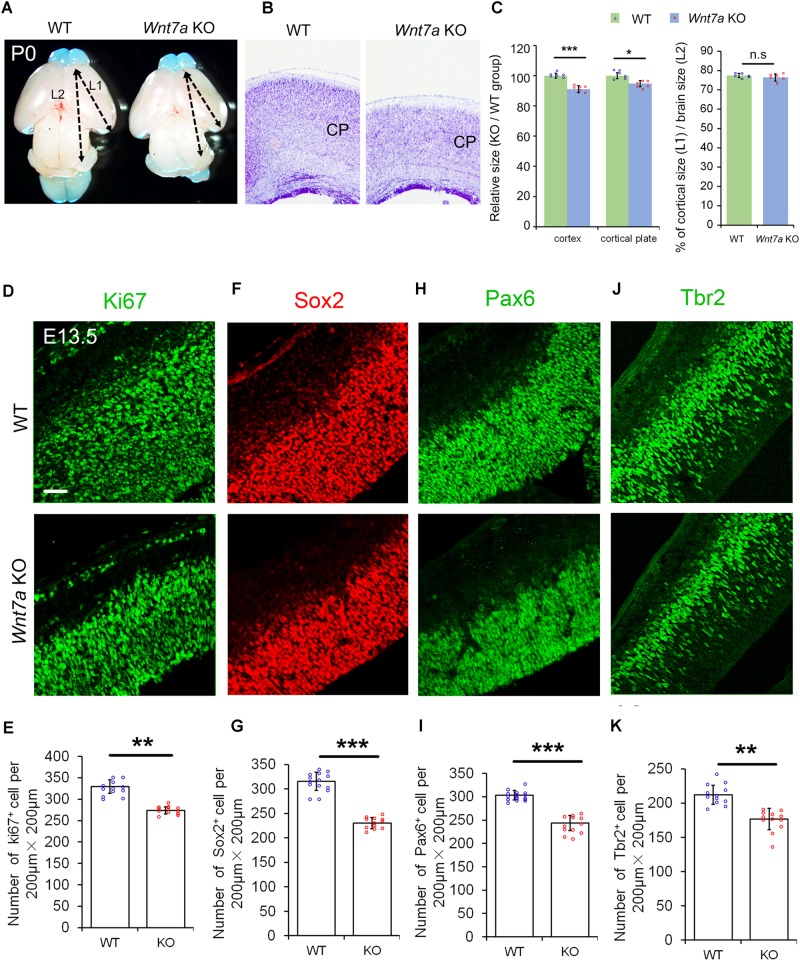
*Wnt7a* positively regulates brain size and proliferation of NPs. **(A)** The cortex of P0 *Wnt7a* knockout (KO) mice was greatly reduced compared to wild type (WT) controls. The arrowheads show the most rostral and caudal regions in the cortex. “L1” represent the cortical length, and “L2” represent the brain length. **(B)** The cortical wall in P0 *Wnt7a* KO mice were thinner than that in WT mice, detected by Nissl staining. CP: cortical plate. **(C)** The relative thickness of the cortex and cortical plate in the KO was normalized from dividing the mean length of *Wnt7a* KO by that of the WT groups. Values of histogram represent mean ± SEM, and each dot represents a data point of the relative thickness in each section or length in the brain images. *n* = 3 brains, at least two sections from each brain. ^∗^*P* < 0.05;^∗∗∗^*P* < 0.001; ns, non-significant; unpaired Student’s *t*-test. **(D–K)** The numbers of Pax6^+^ and Tbr2^+^ neural progenitors were greatly reduced in the E13.5 *Wnt7a* KO cortex. Values of histogram represent mean ± SEM, and each dot represents a data point of the counting number in each section (200 μm bin). *n* = 3 brains, at least four sections from each brain. ^∗∗^*P* < 0.01; ^∗∗∗^*P* < 0.001; unpaired Student’s *t*-test). Scale bar: 50 μm.

Because of *Wnt7a* expression in the cortical VZ, we investigated whether *Wnt7a* regulates NP proliferation by analyzing cortical development in *Wnt7a* knockout mice (*Wnt7a* KO). The body size of *Wnt7a* KO was indistinguishable from that of WT mice. The cortical size and brain size were measured at P0, P5, and P20 (**Figures 2A–C** and Supplementary Figure S2). Compared to WT, the cortical size and brain size of *Wnt7a* KO mice were greatly reduced from P0 to P20, suggesting a progressive brain deterioration (**Figures 2A–C** and Supplementary Figure S2). Moreover, the thickness of the cortical wall was significantly reduced in the brain sections with Nissl staining in *Wnt7a* KO mice (**Figures 2B,C**). Interestingly, the ratios of cortical size versus brain size were similar between WT and KO, suggesting that the overall brain size is reduced in *Wnt7a* KO mice (**Figure 2C** and Supplementary Figure S2).

We then examined whether the NP population was changed in E13.5 *Wnt7a* KO mice using immunohistochemistry. NPs can be detected by labeling cells in the G1, S, G2, and M phases using the anti-Ki67 antibody. The number of Ki67^+^ cells was significantly decreased in the E13.5 *Wnt7a* KO cortex, compared to the control (**Figures 2D,E**). The numbers of Sox2^+^ and Pax6^+^ radial glial cells (RGCs), and Tbr2^+^ IPs were also reduced, suggesting an early reduction of NPs (**Figures 2F–K**). Moreover, because Pax6^+^/Tbr2^+^ cells are under transition from RGCs to IPs, we quantified the number of Pax6^+^/Tbr2^+^ cells. While a significant decrease in the number of Pax6^+^/Tbr2^+^ cells was detected in E13.5 *Wnt7a* KO cortex, the percentages of Pax6^+^/Tbr2^+^ cells versus total Pax6^+^ cells and Pax6^+^/Tbr2^+^ cells versus total Tbr2^+^ cells were unchanged, indicating that *Wnt7a* deletion doesn’t affect transition of RGCs to IPs (Supplementary Figures S3A–D). In addition, even though the total number of Tbr2^+^ cells was reduced, the percentage of Tbr2^+^ cells versus total DAPI^+^ cells remained the same in WT and *Wnt7a* KO cortices, suggesting that reduction in IPs is in proportion with that of total cells (Supplementary Figures S3E,F).

**FIGURE 3:**
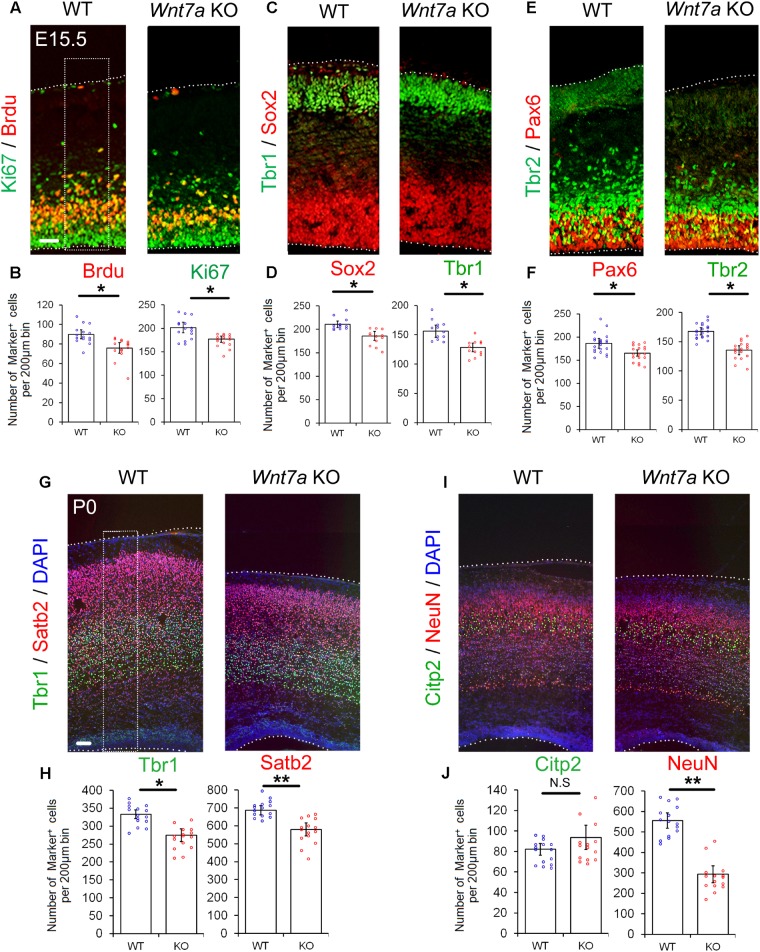
*Wnt7a* promotes neurogenesis at E15.5 and P0. **(A–F)** Compared to controls (WT), *Wnt7a* knockout (KO) cortices at E15.5 displayed a reduction in the numbers of BrdU^+^, Ki67^+^, Sox2^+^, Tbr1^+^, Pax6^+^, and Tbr2^+^ cells. The dashed box represents the cell counting area. Values of histogram represent mean ± SEM, and each dot represents a data point of the counting number in each section (200 μm bin). *n* = 3, at least four sections from each brain. ^∗^*P* < 0.05; unpaired Student’s *t*-test. **(G–J)** In P0 *Wnt7a* KO cortices, the numbers of Tbr1^+^ and Stab2^+^ neurons were greatly reduced. NeuN^+^ neurons but not Citp2^+^ neurons were also reduced. The dashed box represents the cell counting area. Values of histogram represent mean ± SEM, and each dot represents a data point of the counting number in each section (200 μm bin). *n* = 3, at least five sections from each brain. ^∗^*P* < 0.05; ^∗∗^*P* < 0.01; ns, non-significant; unpaired Student’s *t*-test. Scale bar: 100 μm.

Next, we examined whether the early loss of NP population is maintained at E15.5. Compared to the controls, the numbers of BrdU^+^, Ki67^+^, Sox2^+^, Tbr1^+^, Pax6^+^, and Tbr2^+^ cells were greatly reduced in E15.5 *Wnt7a* KO cortices, suggesting that the deletion of *Wnt7a* causes a progressive loss of NPs (**Figures 3A–F** and Supplementary Figures S4A,B).

Because the overall organization of cortical layers is becoming clear, and neuronal production is evident at P0, P0 pups were collected to analyze brain phenotypes without sacrifice of the mother. We examined the expression of Tbr1 (layer VI), Ctip2 (layer V) and Satb2 (layer II, III, and IV) in P0 *Wnt7a* KO and control cortices (Molyneaux et al., 2007). The relative positioning of layer markers in the CP was similar to that of the WT, suggesting that overall cortical layer organization is not greatly affected by *Wnt7a* deletion (**Figures 3G,I**). Despite concordance of the position of layer markers, each layer examined was thinner in the *Wnt7a* KO cortex than that in the control, with significantly fewer mature NeuN^+^ neurons found, and great reductions in the number of Tbr1^+^ and Satb2^+^ neurons (**Figures 3G–J**). The Citp2^+^ neurons showed no appreciable decrease in *Wnt7a* KO mice (**Figures 3I,J**). Moreover, the percentages of Tbr1^+^ and Satb2^+^ cells versus DAPI^+^ cells were unchanged in WT and KO cortices, indicating that the reduction in newborn neurons is in proportion with that of total cells (Supplementary Figure S4C).

Taken together, our results indicate that knockout of *Wnt7a* causes reduced NPs and production of newborn neurons.

### *Sfrp1* Negatively Regulates Proliferation of NPs

**FIGURE 4:**
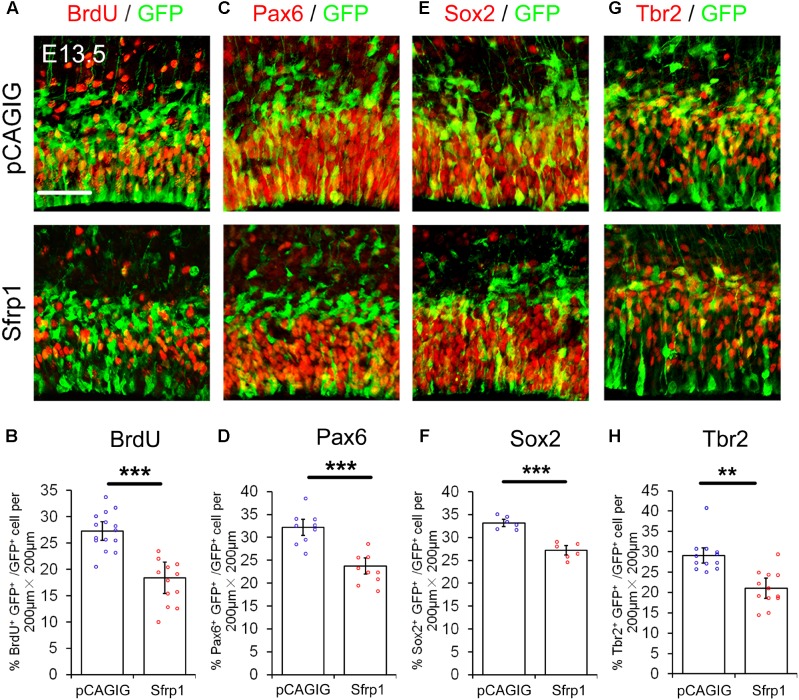
Sfrp1 negatively regulates proliferation of NPs at E13.5. **(A,C,E,G)** Overexpression of *Sfrp1* in E12.5 cortices using *in utero* electroporation, analyzed at E13.5, caused the reduction of BrdU^+^/GFP^+^, Pax6^+^/GFP^+^, Sox2^+^/GFP^+,^ and Tbr2^+^/GFP^+^ neural progenitors. **(B,D,F,H)** The proportion of cells labeled with individual progenitor markers and GFP versus cells labeled with GFP was quantified. Values represent mean ± SEM, and each dot represents a data point of the marker^+^ GFP^+^/GFP^+^ % in each section (200 μm × 200 μm). *n* = 3, at least two sections from each brain. ^∗∗^*P* < 0.01; ^∗∗∗^*P* < 0.001; unpaired Student’s *t*-test. Scale bar: 50 μm.

We next examined whether altering *Sfrp1* expression in the cortex has a similar or an opposite effect on NPs as deleting *Wnt7a* expression. The full length cDNA for *Sfrp1* was cloned (*pCAGIG-Sfrp1*) and was ectopically expressed in E12.5 cortices by using IUE, and embryos were analyzed after 24 h. Overexpression of *Sfrp1* resulted in a decreased number of GFP^+^ NPs that are double-positive for BrdU^+^, Pax6^+^, Sox2^+^ and Tbr2^+^, compared to those of electroporation of the control (*pCAGIG*) in E13.5 cortices, suggesting a decrease of NPs after Sfrp1 overexpression (**Figure 4**).

To test whether the endogenous *Sfrp1* limits the NP numbers *in vivo*, we used *shRNA* designed to outcompete endogenous *Sfrp1* transcripts. The *Sfrp1* knockdown efficiency were verified in mouse Neuro2A cell by qRT-PCR (Supplementary Figure S5). The construct of *shRNA* (*Sfrp1-sh4*) that shows the highest knockdown efficiency among four tested shRNAs was used to perform IUE. Greater proportions of GFP^+^ NPs expressed BrdU, Pax6 and Sox2 were found in the VZ/SVZ following electroporation of the *Sfrp1-sh4* (Supplementary Figures S6A–F). Tbr2^+^ NPs displayed no appreciable increase (Supplementary Figures S6G,H). These results indicate that *Sfrp1* negatively modulates NP proliferation.

### *Sfrp1* Has an Opposite Role of *Wnt7a* in Regulating NP Proliferation

Based on opposite effect of *Wnt7a* and *Sfrp1* on NP development, we suspected that Wnt7a might be regulated by its antagonists during cortical development. Previous studies have shown that *Dkk1* is an antagonist of *Wnt7a* (Fortress et al., 2013). To examine how the *Wnt7a* antagonist may regulate NP development in the cortex, we over-expressed both *Wnt7a* and *Dkk1* in the VZ of cortex using IUE. While *Wnt7a* promoted expansion of NPs, as shown by an increased number of BrdU^+^ and Pax6^+^ cells, over-expression of *Dkk1* and *Wnt7a* in the VZ dampened *Wnt7a* effects, suggesting an antagonistic regulation of Dkk1 (Supplementary Figure S7). Moreover, increasing *Dkk1* dosage caused a greater decrease in the number of BrdU^+^ and Pax6^+^ cells, suggesting a dosage-dependent antagonistic regulation of *Dkk1* on *Wnt7a* (Supplementary Figure S7).

**FIGURE 5:**
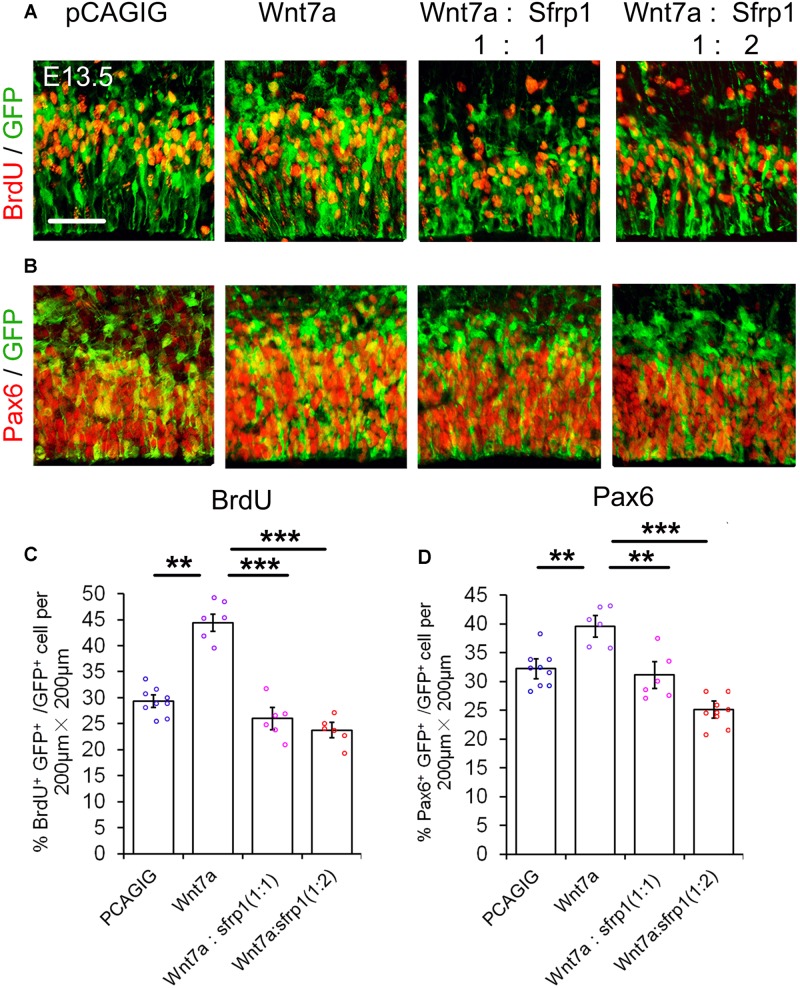
Sfrp1 suppresses *Wnt7a* activity in neural progenitor proliferation dosage-dependent manner. **(A,B)** Co-expression of *Sfrp1* and *Wnt7a* dampened the effect of *Wnt7a* in expanding neural progenitors at E13.5. **(C,D)** The numbers in BrdU^+^/GFP^+^ and Pax6^+^/GFP^+^ neural progenitors showed a decreasing trend with a proportional increase of *Sfrp1* (*Wnt7a*:*Sfrp1* = 1:1 vs. *Wnt7a*:*Sfrp1* = 1:2). Values represent mean ± SEM, and each dot represents a data point of the marker^+^ GFP^+^/GFP^+^ % in each section (200 μm × 200 μm). *n* = 3, at least two sections from each brain. ^∗∗^*P* < 0.01; ^∗∗∗^*P* < 0.001; unpaired Student’s *t*-test. Scale bar = 50 μm.

If *Sfrp1* also has the functions as a *Wnt7a* antagonist, it should have a similar effect to *Dkk1* in NP development. With this in mind, *Wnt7a* and *Sfrp1* were both overexpressed in the cortex using IUE. Similar to *Dkk1*, *Wnt7a-Sfrp1* overexpressed in the VZ caused a reduction of BrdU^+^ and Pax6^+^ cells (**Figure 5**). Moreover, increasing the dosage of *Sfrp1* had a more profound activity in suppressing *Wnt7a* effect on NP expansion (**Figure 5**).

Our results suggest that similar to *Dkk1*, *Sfrp1* acts as an antagonist of *Wnt7a* and negatively regulates expansion of NPs.

### *Sfrp1* Inhibits *Wnt7a* Activity in *TOPflash Luciferase* Reporter Assay

**FIGURE 6:**
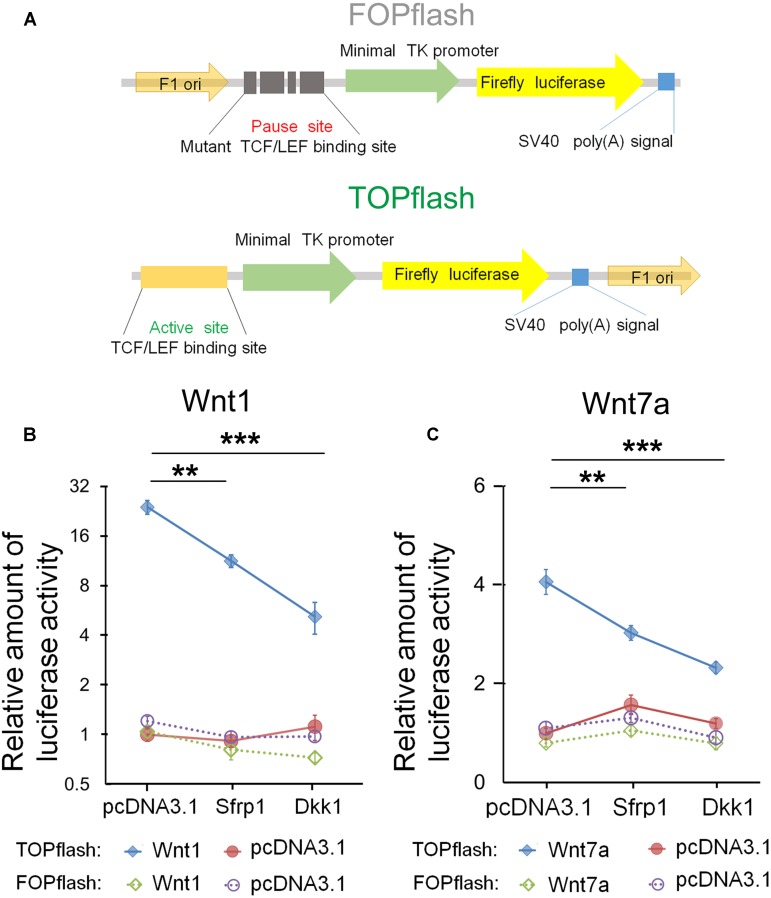
Sfrp1 inhibits *Wnt7a* activity in the *TOPflash luciferase* reporter assay. **(A)** *TOPflash* is a *luciferase* reporter of β-catenin-mediated transcriptional activation with active TCF/LEF binding sites, which affect the *firefly luciferase* expression. The control plasmid is *FOPflash*, which contains mutant TCF/LEF binding sites. **(B,C)** After transfection of the *pcDNA3.1-Sfrp1* and *pcDNA3.1-Dkk1*, a statistically significant decrease in *luciferase* activity of *Wnt1* and *Wnt7a* was observed in comparison with controls. Values represent mean ± SEM. *n* = 3, ^∗∗^*P* < 0.01; ^∗∗∗^*P* < 0.001; unpaired Student’s *t*-test.

Based on the dosage-dependent regulation of *Sfrp1* on *Wnt7a*, we tested whether *Sfrp1* could down-regulate the *Wnt7a* activity. To validate *Sfrp1-Wnt7a* interaction, we used the *TOPflash luciferase* reporter assay containing the active TCF/LEF binding sites, which is the classical method to identify canonical Wnt/β-catenin activity (**Figure 6A**) (Veeman et al., 2003). If the canonical Wnt signaling is activated, the β-catenin will be associated with the TCF/LEF transcription factors to promote the *Firefly luciferase* activity. The mutant TCF/LEF binding site of *FOPflash* was used as the control (**Figure 6A**).

Wnt1 is a known molecule of the Wnt signaling and is crucial for early development of the CNS (Leal et al., 2011; Cai et al., 2013). As the positive control, we first tested whether *Dkk1* and *Sfrp1* can block *Wnt1* in Neuro2A cells. Compared to the *FOPflash* group, the *luciferase* activity of *Wnt1* in *Dkk1* overexpression treatment was significantly decreased in the *TOPflash* group (**Figure 6B**). Agreed with *Dkk1*, the *luciferase* activity of *Sfrp1* overexpression showed a similar decrease (**Figure 6B**).

Next, we tested whether *Sfrp1* can inhibit *Wnt7a* in a similar fashion to how Wnt1 is negatively regulated in the aforementioned experiment. We found that the *luciferase* activity of *Wn7a* was decreased appreciably in both *Sfrp1* and *Dkk1* over-expression treatment, suggesting that *Sfrp1* acts like the known antagonist *Dkk1*, and blocks the *Wnt7a* signal (**Figure 6C**).

In summary, Sfrp1 has an attenuating role in Wnt signaling by blocking *Wnt1* and Wnt7a *in vitro*.

## Discussion

The maintenance of normal cortical formation and size is essential for brain function. The Wnt signaling plays critical roles to regulate cell cycle control, neuronal differentiation and tissue repair (Chenn and Walsh, 2003; Kalani et al., 2008; Piccin and Morshead, 2011; Delaunay et al., 2014). The precise antagonistic regulation of Wnt members by Wnt modulators also controls cortical neurogenesis. Our study shows that *Wnt7a* and *Sfrp1* are co-expressed in cortical NPs and their opposite role is essential for controlling NP expansion and neuronal production.

Among the many signals known to influence the CNS development, the Wnt signal has attracted great attention. Wnt/β-catenin signaling acts upstream of a complex and dynamic temporal network to control progenitor fate (Draganova et al., 2015): long-term overexpression of *Wnt3a* leads to cortical dysplasia by inducing early differentiation of IPs into neurons and the heterotopias of these newborn neurons (Munji et al., 2011). Studies have shown the role of *Wnt7a* in axon development and guidance, as well as synapse formation and maintenance (Hall et al., 2000; Cerpa et al., 2008; Ciani et al., 2011, 2015). Investigations of *Wnt7* in the early step of neurogenesis in the cerebral cortex have just begun (Qu et al., 2013; Long et al., 2016). Transcriptome sequencing data from us and others have shown that *Wnt7b, Wnt7a*, and *Wnt5a* are the most abundant Wnt factors in the E12.5, E16.5, and E17.5 cortices (Wang et al., 2016; Nguyen et al., 2018). Moreover, we have found that *Wnt7a* is highly expressed in the VZ and *Wnt7b* in the intermediate zone and CP, which is consistent with the RNA-seq results from isolating specific cellular zones and layers in E14.5 and E15.5 cortices (Ayoub et al., 2011; Belgard et al., 2011; Aprea et al., 2013; Liu et al., 2016). How distinct expression patterns of different Wnts are established in developing cortices remains unclear. Differential expression of Wnt7a and Wnt7b in the cortical layers may determine their different roles in cortical neurogenesis (Stenman et al., 2008; Durak et al., 2016): *Wnt7a* promotes neurogenesis by regulating genes involved in cell cycle control and neuronal differentiation (Qu et al., 2013); the increased *Wnt7b* modulates neuronal differentiation by regulating T-domain transcription factors Tbr1 and Tbr2 (Papachristou et al., 2014).

Moreover, we have shown that the deletion of *Wnt7a* expression causes microcephaly by reducing the population of NPs and newborn neurons. These data are consistent with previous reports demonstrating that *Wnt7a* positively regulates NPs and neurogenesis (Qu et al., 2013; Long et al., 2016; Wang et al., 2016). Recent research has shown that *Wnt7a* regulates the asymmetry of spindles in neuroepithelial cells in the VZ, which is linked to asymmetric cell division (Delaunay et al., 2014). The embryonic ventral midbrain of *Wnt7a* KO mice displays reduced Sox2^+^ progenitors (Fernando et al., 2014). We have also found that Sox2^+^ progenitors are decreased in the cerebral cortex at E13.5. Decreased expansion of cortical NPs is likely a major cause of microcephaly in *Wnt7a KO* mice. Among Wnt molecules, Wnt7a is a known regulator in the beta-catenin signal pathway (mmu04310) functioning in different biological processes (Daneman et al., 2009; Ciani et al., 2011; Qu et al., 2013; King et al., 2015). Wnt molecules are associated with Hippo signaling pathway, Integrin signaling and Notch signaling (Qu et al., 2013; Ciani et al., 2015; Wang et al., 2016). These pathways likely cooperate to regulate cortical development.

Sfrps are a family of receptors known to possess a Wnt-binding frizzled CRD, and abnormal expression of *Sfrp1* leads to CNS functional disorders (Esteve et al., 2011, 2018). *Sfrp1* is a key member of the Sfrp family that can bind directly to Wnts via their regions of homology to Fz. In the CNS, *Sfrp1* can block dopamine neuron development, dendritic development and hippocampus formation (Rosso et al., 2005; Miquelajauregui et al., 2007; Kele et al., 2012). In this study, we have found that *Sfrp1* is expressed in the VZ of the mouse embryonic cerebral cortex, which is consistent with the observation of its expression restricted to the proliferative zone in the CNS (Augustine et al., 2001). Similar to the known antagonist Dkk1, we have found that overexpression of *Sfrp1* reduces the NP population, and *Sfrp1* significantly decreases the number of NPs in a dosage-dependent manner, suggesting an opposite role of Sfrp1 in cortical development compare to Wnt7a (Adamska et al., 2004; Kim et al., 2008; Osada et al., 2010). In the recent study of *Sfrp1* knockout mice, the authors observed an increase in the number of BrdU^+^/Tbr2^+^ cells in E12.5 Sfrp1^-/-^ cortex (Esteve et al., 2018). We think that the reason we did not detect an increase of Tbr2^+^ cells when Sfrp1 is knocked down, it is likely due to the efficiency of shRNA of Sfrp1, compared to the gene knockout. Moreover, recent studies have shown that Sfrps interact with the Wnt signaling, Hedgehog signaling, BMP and Notch signaling (Katoh and Katoh, 2006; Mii and Taira, 2009; Misra and Matise, 2010; Esteve et al., 2011, 2018). It is likely a combined effort of Sfrp1 with other signals contributes to cortical development.

Sfrps is a physiological Wnt-signaling scavenger that binds directly to Wnts due to their similarity to the receptor Frizzled, thus, it is capable of regulating the availability of Wnt proteins (Finch et al., 1997; Rattner et al., 1997; Baarsma et al., 2013; Cruciat and Niehrs, 2013). The exclusive repression of the Wnt pathway is possible by selective Sfrps in cortical development (Mikels and Nusse, 2006; Lacour et al., 2017). *Sfrp1* and *Sfrp3* are expressed in opposing anterolateral to caudomedial gradients, and regulate normal temporal advancement of neuronal birth and maturation in anterior and lateral cortical regions by selectively modulating Wnts (Kim et al., 2001b). Previous studies have shown that Dkks inhibit the canonical Wnt pathway by internalizing LRP5/6, whereas Sfrps inhibit both the canonical and non-canonical pathways by binding Wnt ligands or Frizzled (Dees et al., 2014; Majchrzak-Celinska et al., 2016). The future study will be to investigate whether Sfrp1 directly binds to Wnt7a or through other mechanisms in the cortex.

The reciprocal control of *Wnt7a* and *Sfrp1* may be a dosage-dependent compensatory mechanism to maintain normal cortical formation during early development. Our study reveals that an optimal expression level of *Wnt7a* and *Sfrp1* is critical for proper establishment of the NP population. Further work will be dedicated to explore the precise regulation of how different Sfrps mediate canonical Wnt signaling pathway in NP proliferation and differentiation during embryonic cortical development. Our findings suggest that dysregulation of the Wnt signaling can lead to developmental defects similar to human cortical malformation disorders such as microcephaly.

## Author Contributions

TS: conceived and designed the experiments. NM, SB, TL, and TM: experiment. NM, SB, SH, ZW, GH, and TS: result analysis. NM and TS: wrote the paper. NM and TS: edited paper.

## Conflict of Interest Statement

The authors declare that the research was conducted in the absence of any commercial or financial relationships that could be construed as a potential conflict of interest.

## Footnotes

1 http://www.ncbi.nlm.nih.gov/geo/

## Abbreviations

CNS: central nervous system
CP: cortical plate
CRD: cysteine-rich domain
E0.5: embryonic day 0.5
Fz: frizzled
IP: intermediate progenitor
ISH: *in situ* hybridization
IUE: *in utero* electroporation
NP: neural progenitor
P0: postnatal day 0
PFA: paraformaldehyde
qRT-PCR: quantitative real-time PCR
RNAi: RNA interference
RT-PCR: reverse transcription-PCR
Sfrp1: secreted frizzled-related protein 1
shRNA: short hairpin RNA
SVZ: subventricular zone
TGF-β: transforming growth factor-β
VZ: ventricular zone
