# Supplementary material for: Opposite Roles of Wnt7a and Sfrp1 in Modulating Proper Development of Neural Progenitors in the Mouse Cerebral Cortex
Source: Front Mol Neurosci. 2018 Jul 17;11:247. doi: 10.3389/fnmol.2018.00247 (PMC6056652; doi:10.3389/fnmol.2018.00247)
Supplement: Supplementary file 5 [file Data_Sheet_1.pdf]

Supplementary materials:

Opposite roles of Wnt7a and Sfrp1 in modulating proper development of neural progenitors in the mouse cerebral cortex

Nan Miao[1, ‡], Shan Bian[2, ‡], Trevor Lee[2], Taufif Mubarak[2], Shi-Ying Huang[3], Zhi-Hong Wen[4], Ghulam Hussain[5], Tao Sun[1, 2, *]

[1]Center for Precision Medicine, School of Medicine and School of Biomedical Sciences, Huaqiao University, Xiamen, Fujian 361021, China

[2]Department of Cell and Developmental Biology, Cornell University Weill Medical College, 1300 York Avenue, Box 60, New York, NY 10065, USA

[3]College of Oceanology and Food Science, Quanzhou Normal University, Quanzhou 362000, China

[4]Marine Biomedical Laboratory and Center for Translational Biopharmaceuticals, Department of Marine Biotechnology and Resources, National Sun Yat-sen University, Kaohsiung 80424, Taiwan

[5]Department of Physiology, Government College University, Faisalabad, Pakistan

[*]Corresponding author: Dr. Tao Sun, Email: taosun@hqu.edu.cn.

[‡] These authors contributed equally to this work

Table

Table. S1 Quality control analysis of RNA-seq result from mouse E12.5 cerebral cortices.

Table. S2 Mapping Statistics of RNA-seq result from mouse E12.5 cerebral cortices.

Table. S3 RNA sequencing reads counts from mouse E12.5 cerebral cortices.

Table. S4 ALL RPKM values of RNA-seq result from mouse E12.5 cerebral cortices.

Figure legend：

Figure. S1 *Wnts* and *Sfrps* expression in mouse E12.5 cortices.

A. RNA sequencing result from mouse E12.5 cortices showed that *Wnt7a*, *Wnt7b* and *Sfrp1* are highly expressed (RPKM >500).

B. In coronal sections of mouse E12.5 cerebral cortices, *Sfrp1* was expressed in the ventricular zone, detected by *in situ* hybridization, and other *Sfrp*s such as Sfrp2, Sfrp4 and Sfrp5 showed low expression.

Figure. S2 *Wnt7a* knockout (KO) mice display microcephaly.

A and **B.** The cortex of *Wnt7a* KO mouse was greatly reduced compared to the wildtype (WT) at P5. “L1” represent the cortical length, and “L2” represent the brain length. The relative size (dividing the mean length of KO by that of the WT groups) of the cortex showed significantly reduction. The percentage of L1/L2 displayed no changes in P5 *Wnt7a* KO and WT mice.

C and **D.** The cortices of *Wnt7a* KO mice were greatly reduced at P20.

Values of histogram represent mean ± S.E.M., and each dot represents a data point of the relative size in each section (200μm bin) and length in each brain image. n=3 brains, each brain has 3 sections. *: $P < 0.05$; N.S: none significance; unpaired Student’s T-test.

Figure. S3 Knockout of *Wnt7a* causes a reduction of Pax6$^+$ and Tbr2$^+$progenitors.

A-D. Compared to wildtype (WT) groups, Pax6$^+$Tbr2$^+$ cells were decreased in E13.5 *Wnt7a* knockout (KO) cortices. The percentage of Pax6$^+$Tbr2$^+$/Pax6$^+$ and Pax6$^+$Tbr2$^+$/Tbr2$^+$ showed no significant changes.

E and **F**. Decreased numbers of intermediate progenitors (IPs) labeled with Tbr2 in E13.5 *Wnt7a* KO cortices as compared to WT. The percentage of Tbr2$^+$/DAPI displayed no significant change between *Wnt7a* KO and WT cortices.

Values of histogram represent mean ± S.E.M., and each dot represents a data point of the counting number in each section (200μm bin). n=3, 5 sections from each brain. *: $P < 0.05$; n.s: none significance; unpaired Student's T-test. Scale bar: 100 μm.

Figure. S4 Knockout of *Wnt7a* causes the reduction of Sox2^{+} progenitors and reduced neurogenesis.

A and **B.** Compared to wildtype (WT) controls, *Wnt7a* knockout (KO) cortices at E15.5 displayed a reduction in the number of Sox2^{+} neural progenitors.

C. The ratio of Tbr1^{+} and Satb2^{+} cells versus DAPI^{+} cells displayed no changes in P0 *Wnt7a* KO and WT cortices.

Values of histogram represent mean ± S.E.M., and each dot represents a data point of the counting number in each section (200μm bin). n=3, at least 4 sections from each brain. **: $P < 0.01$; n.s: none significance; unpaired Student's t test. Scale bar = 50 μm.

Figure. S5 *Sfrp1* shRNA efficiency.

Sfrp1-sh4 showed the highest knockdown efficiency in 4 different shRNAs for *Sfrp1*. ***: $P < 0.001$; unpaired Student's t test.

Figure. S6 Knockdown of *Sfrp1* promotes expansion of neural progenitors.

A, C, E and **G.** Knockdown of *Sfrp1* in E13.5 cortices using *in utero* electroporation, analyzed at E14.5, caused the reduction of BrdU^{+}/GFP^{+}, Pax6^{+}/GFP^{+}, Sox2^{+}/GFP^{+} and Tbr2^{+}/GFP^{+} neural progenitors.

B, D, F and **H.** The proportion of cells labeled with individual progenitor markers and GFP versus cells labeled with GFP was quantified.

Values of histogram represent mean ± S.E.M., and each dot represents a data point of the marker^{+} GFP^{+}/ GFP^{+} % in each section (200 × 200 μm). n=3, at least 4 sections from each brain. *: $P < 0.05$; n.s: none significance; unpaired Student's t test. Scale bar = 50μm.

Figure. S7 *Dkk1* is a known antagonist of *Wnt7a* in proliferation of neural progenitors in a dosage-dependent manner.

A and **B.** Co-expression of *Dkk1* and *Wnt7a* dampened the effect of *Wnt7a* in expanding neural progenitors.

C and **D.** The numbers in $BrdU^+/GFP^+$ and $Pax6^+/GFP^+$ neural progenitors showed a decreasing trend with a proportional increase of *Dkk1* (*Wnt7a* : *Dkk1*=1:1 vs *Wnt7a* : *Dkk1*=1:2).

Values of histogram represent mean ± S.E.M., and each dot represents a data point of the $marker^+ GFP^+/ GFP^+$ % in each section (200 × 200 μm). n=3, at least 2 sections from each brain. *: $P < 0.05$; **: $P < 0.01$; ***: $P < 0.001$; n.s: none significance; unpaired Student's t test. Scale bar = 50μm.

Figure. S1

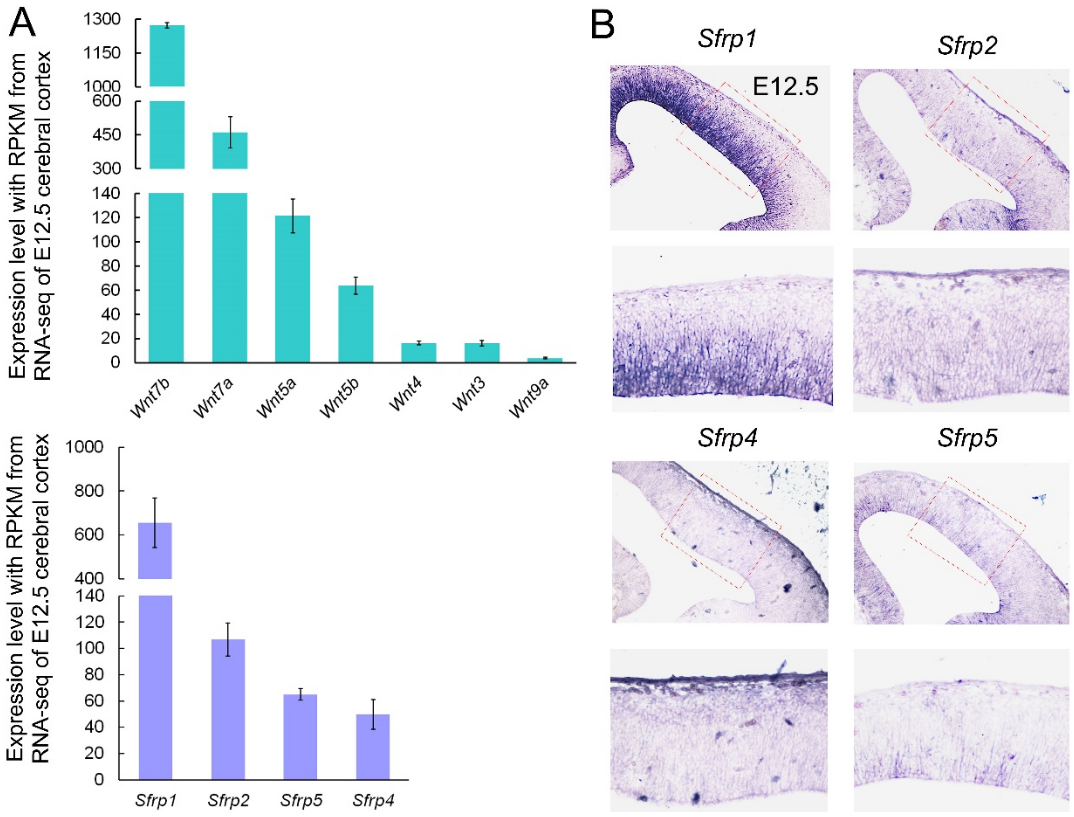
A
Expression level with RPKM from RNA-seq of E12.5 cerebral cortex
1300
1150
1000
600
450
300
140
120
100
80
60
40
20
0
Wnt7b
Wnt7a
Wnt5a
Wnt5b
Wnt4
Wnt3
Wnt9a
Expression level with RPKM from RNA-seq of E12.5 cerebral cortex
1000
800
600
400
140
120
100
80
60
40
20
0
Sfrp1
Sfrp2
Sfrp5
Sfrp4
B
Sfrp1
Sfrp2
E12.5
Sfrp4
Sfrp5

Figure. S2

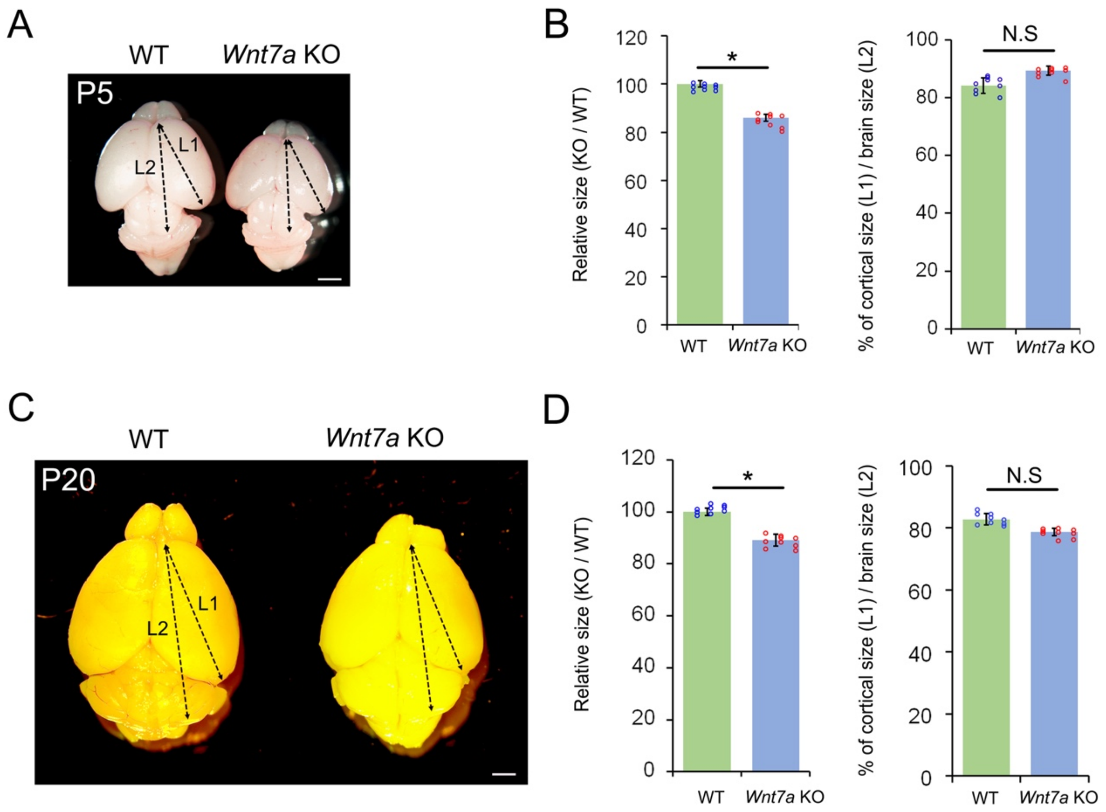
A
WT
Wnt7a KO
P5
L1
L2
B
Relative size (KO / WT)
120
100
80
60
40
20
0
*
WT
Wnt7a KO
% of cortical size (L1) / brain size (L2)
N.S
C
WT
Wnt7a KO
P20
L1
L2
D

Figure. S3

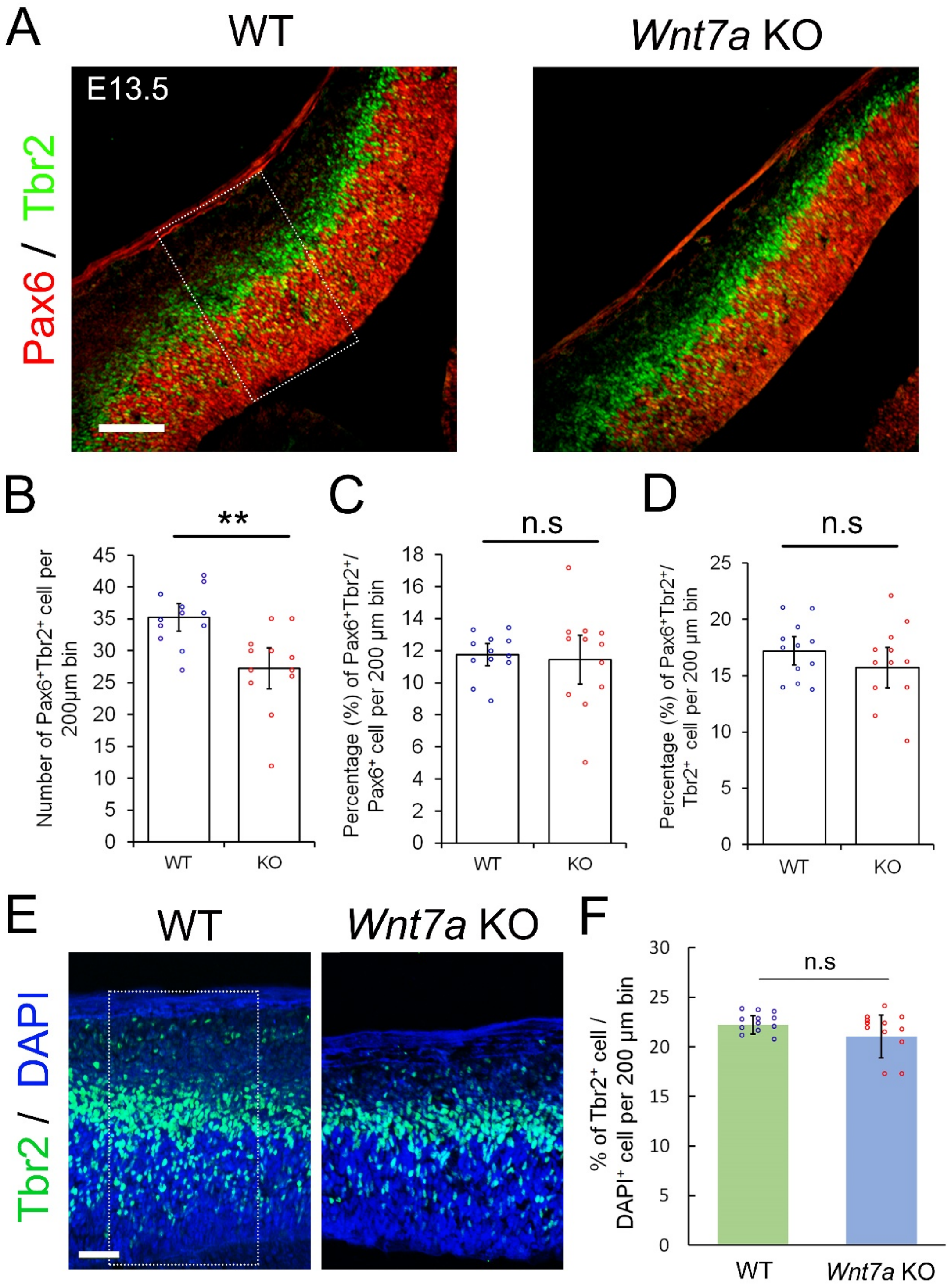

A
WT
Wnt7a KO
E13.5
Pax6 / Tbr2
B
**
Number of Pax6+Tbr2+ cell per 200µm bin
45
40
35
30
25
20
15
10
5
0
WT
KO
C
n.s
Percentage (%) of Pax6+Tbr2+/ Pax6+ cell per 200 µm bin
18
16
14
12
10
8
6
4
2
0
WT
KO
D
n.s
Percentage (%) of Pax6+Tbr2+/ Tbr2+ cell per 200 µm bin
25
20
15
10
5
0
WT
KO
E
WT
Wnt7a KO
Tbr2 / DAPI
F
n.s
% of Tbr2+ cell / DAPI+ cell per 200 µm bin
30
25
20
15
10
5
0
WT
Wnt7a KO

Figure. S4

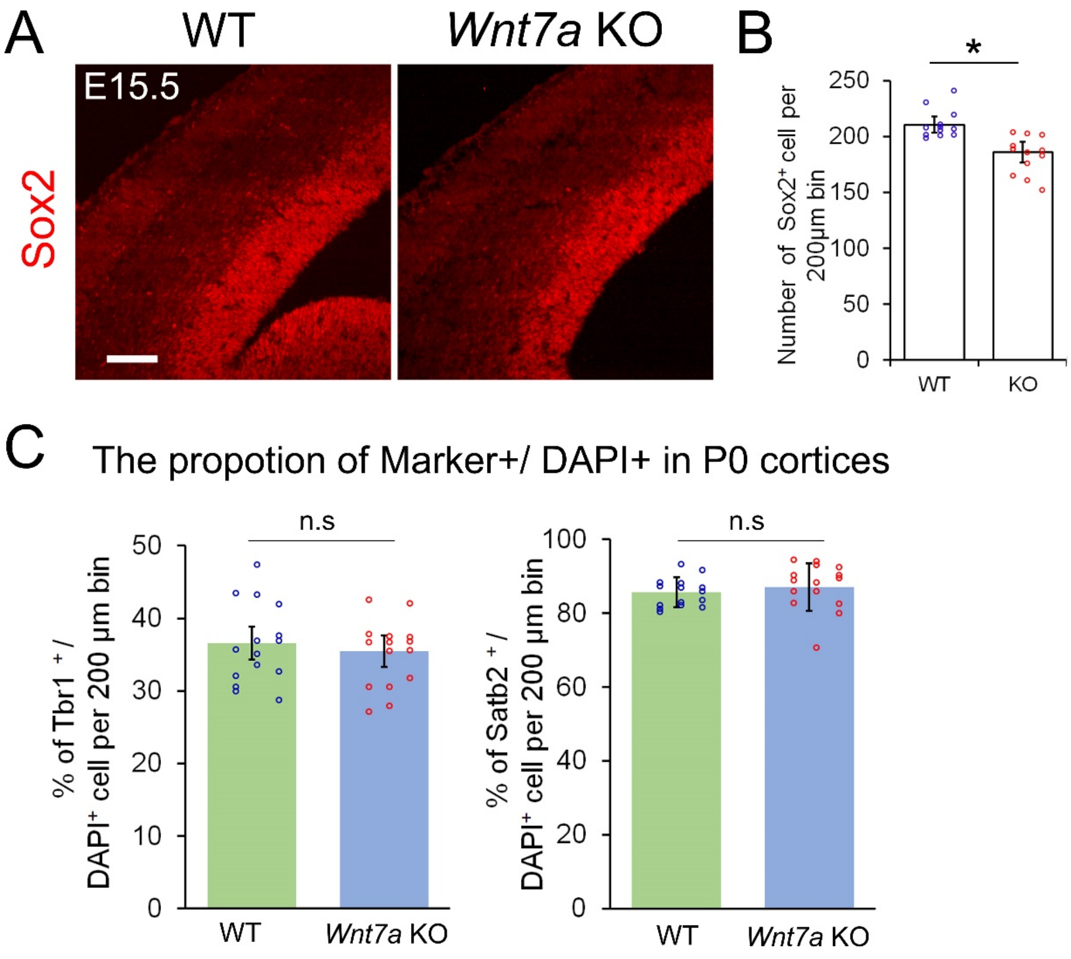
A
WT
Wnt7a KO
E15.5
Sox2
B
*
Number of Sox2+ cell per 200μm bin
250
200
150
100
50
0
WT
KO
C
The propotion of Marker+/ DAPI+ in P0 cortices
n.s
% of Tbr1 + / DAPI+ cell per 200 μm bin
50
40
30
20
10
0
WT
Wnt7a KO
n.s
% of Satb2 + / DAPI+ cell per 200 μm bin
100
80
60
40
20
0
WT
Wnt7a KO

Figure. S5

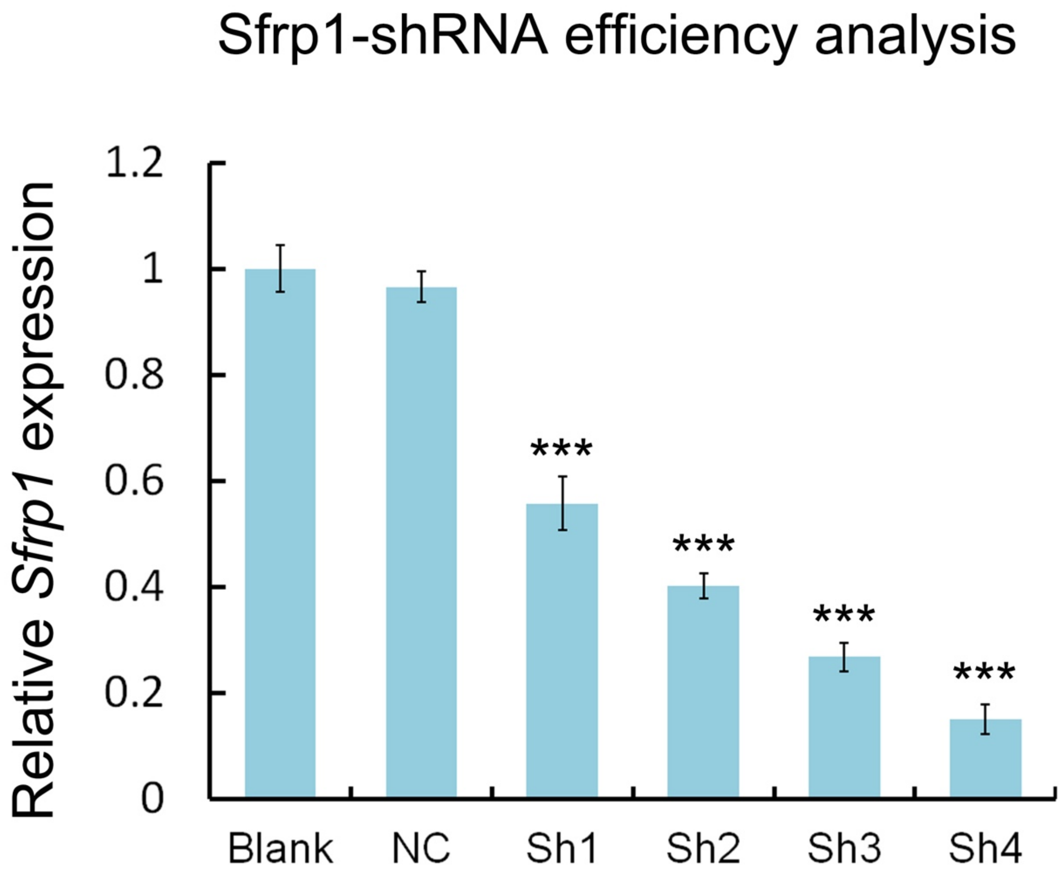
Sfrp1-shRNA efficiency analysis
Relative *Sfrp1* expression
1.2
1
0.8
0.6
0.4
0.2
0
***
***
***
***
Blank
NC
Sh1
Sh2
Sh3
Sh4

Figure. S6

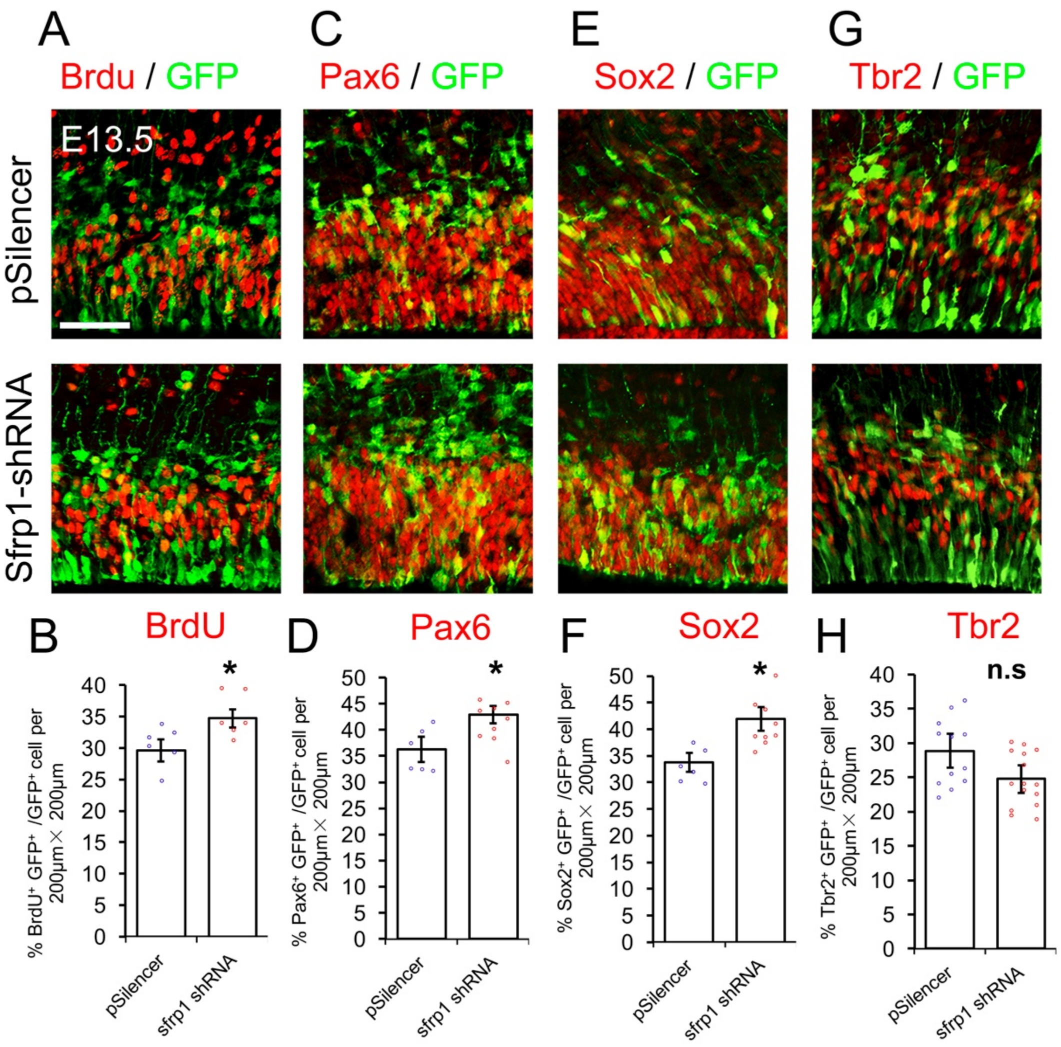
A
Brdu / GFP
C
Pax6 / GFP
E
Sox2 / GFP
G
Tbr2 / GFP
E13.5
pSilencer
Sfrp1-shRNA
B
BrdU
% BrdU+ GFP+ /GFP+ cell per 200µm× 200µm
pSilencer
sfrp1 shRNA
*
D
Pax6
% Pax6+ GFP+ /GFP+ cell per 200µm× 200µm
*
F
Sox2
% Sox2+ GFP+ /GFP+ cell per 200µm× 200µm
*
H
Tbr2
% Tbr2+ GFP+ /GFP+ cell per 200µm× 200µm
n.s

Figure. S7

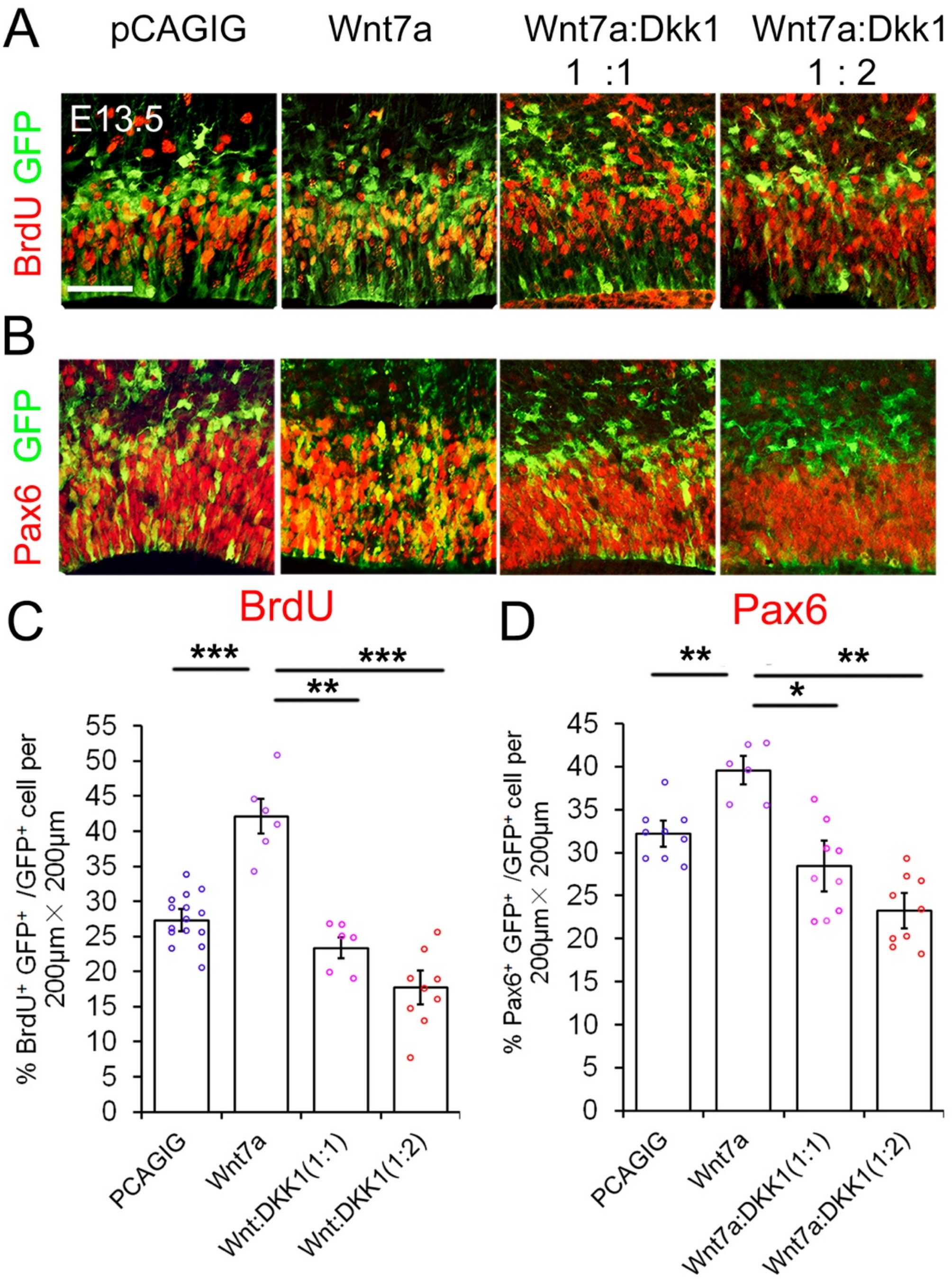
A
pCAGIG
Wnt7a
Wnt7a:Dkk1
1 :1
Wnt7a:Dkk1
1 : 2
E13.5
BrdU GFP
B
Pax6 GFP
C
BrdU
***
***
**
% BrdU⁺ GFP⁺ /GFP⁺ cell per 200μm × 200μm
PCAGIG
Wnt7a
Wnt:DKK1(1:1)
Wnt:DKK1(1:2)
D
Pax6
**
**
*
% Pax6⁺ GFP⁺ /GFP⁺ cell per 200μm × 200μm
PCAGIG
Wnt7a
Wnt7a:DKK1(1:1)
Wnt7a:DKK1(1:2)
